# Children with autism spectrum disorder present glymphatic system dysfunction evidenced by diffusion tensor imaging along the perivascular space

**DOI:** 10.1097/MD.0000000000032061

**Published:** 2022-12-02

**Authors:** Xin Li, Cailian Ruan, Abdoulaye Issotina Zibrila, Mazen Musa, Yifan Wu, Zhengxiang Zhang, Heng Liu, Mustafa Salimeen

**Affiliations:** a Department of Anaesthesiology, School of Medicine, Yan’an University, Yanan, China.; b Anatomy Department, School of Medicine, Yan’an University, Yanan City, China; c Laboratory of Experimental Pharmacology, Department of Animal Physiology, Faculty of Science and Technology, University of Abomey-Calavi, Abomey-Calavi, Benin; d Department of Orthodontics, Al Tegana Dental Teaching Hospital, Faculty of Dentistry, University of Science and Technology, Omdurman, Sudan; e MD Undergraduate Program, School of Medicine, Yan’an University, Yan’an City, China; f Department of Pharmacology, School of Medicine, Yan’an University, Yan’an City, China; g Department of Radiology, Affiliated Hospital of Zunyi Medical University, Medical Imaging Center of Guizhou Province, Zunyi City, China; h Department of Radiology, Affiliated Hospital, School of Medicine, Yan’an University, Yan’an City, China; i Department of Radiology, Dongola Teaching Hospital Faculty of Medicine and Health Sciences, University of Dongola, Dongola, Republic of Sudan, Dongola, Sudan.

**Keywords:** autism spectrum disorder, diffusion tensor imaging, glymphatic system, magnetic resonance imaging

## Abstract

This study used diffusion tensor imaging (DTI) along the perivascular space (DTI-ALPS) to assess glymphatic system function in autism spectrum disorder (ASD) compared to healthy controls. Patients with ASD may have glymphatic system dysfunction, which is related to age. We retrospectively included 30 children with ASD and 25 healthy controls in this study. 3T magnetic resonance imaging scanner was used to perform DTI magnetic resonance imaging on all participants, and the DTI-ALPS index was calculated from the DTI data. Additionally, we evaluated how the DTI-ALPS index differed between the 2 groups. Moreover, we examined the relationships between the bilateral DTI-ALPS index and the age of the participants. The DTI-ALPS index considerably differed between groups. In the left index (1.02 ± 0.12 vs. 1.27 ± 0.25, *P* < .001) and in the right index (1.03 ± 0.12 vs. 1.32 ± 0.20, *P* < .001), the DTI-ALPS in ASD patients was significantly lower than that in healthy controls. Furthermore, the DTI-ALPS index was strongly and positively associated with age. In patients with ASD, there is a glymphatic system dysfunction. This is intimately correlated to age. Our findings suggest the importance of the DTI-ALPS approach in assessing the function of the glymphatic system in ASD.

## 1. Introduction

The glymphatic system is a well-organized system for transporting fluids.^[1]^ The aquaporin-4 water channels allow the flow of cerebrospinal fluid (CSF) into the brain through the perivascular spaces of the leptomeningeal and penetrating arteries and then into the brain interstitial fluid (ISF). The channel then guides flow to the venous perivascular and perineuronal regions, where it eventually reaches the lymphatic drainage arteries of the meninges and cervical spine.^[1]^ The glymphatic system is a vital system for removing protein waste products from the brain.^[2]^ CSF removes waste proteins from the tissue as it reaches the interstitial space.^[3,4]^ The CSF-ISF fluid, mixed with interstitial waste solutes, is then carried to the large central veins’ perivenous compartment, where it exits into lymphatic vessels and the systemic circulatory system.^[3,5,6]^ The glymphatic system is more activated during slow-wave sleep than during awake.^[4,7]^

Brain section microscopy, 2-photon microscopy, transcranial mesoscopic imaging, and proton emission tomography-magnetic resonance imaging (MRI) have all been used to visualize the glymphatic system.^[8–11]^ Clinical imaging of the glymphatic system in humans is a new field among these imaging techniques. Recently, several novel approaches for evaluating the glymphatic system in humans have been developed. Brain MRI is an excellent technique for whole-brain research since it can reveal deep brain regions.^[8]^

Diffusion tensor imaging (DTI) along the perivascular space (DTI-ALPS) has been proposed as a glymphatic means product.^[11]^ It measures the diffusivity using the diffusion tensor method to determine the mobility of water molecules in the direction of the perivascular space. It is based on the fact that the perivascular space, as well as the medullary veins, are orthogonal to the projection and association fibers at the level of the lateral ventricle body. The projection and association fibers will be affected by glymphatic system malfunction with histological alterations.^[11,12]^ Because the DTI-ALPS approach is noninvasive and does not require gadolinium-based contrast delivery, it can be used in routine clinical settings.^[11,13]^

Given the novelty of the glymphatic system concept, a growing number of studies have been published to better understand the role of the glymphatic system in neurological disorders such as sleep behavior disorder, diabetes, temporal lobe epilepsy, Alzheimer’s disease, Parkinson’s disease, normal pressure hydrocephalus, and other neurological illnesses.^[11,14–19]^ Furthermore, the DTI-ALPS approach has never been used to investigate the function of the glymphatic system in individuals with autism spectrum disorder (ASD).

ASD is a complex, heterogeneous neurodevelopmental disease characterized by repetitive activities, constrained interests, and impairments in social communication and reciprocity.^[20]^ In early childhood, when the symptoms first appear, the illness is typically assumed to last a lifetime. For many years, autism was believed to be a rare condition after it was first identified in 1943 by Leo Kanner. However, during the last few decades, the estimated prevalence has substantially and persistently climbed. This is believed to be the result of several variables, such as the expansion of illness mechanisms, some novel pathophysiological detection, and increasing awareness and knowledge of the disorder.^[20]^ There might also be more factors which are currently unknown and subjected to much conjecture. The Centers for Disease Control and Prevention (CDC) currently estimated that 1 in 68 children are diagnosed with ASD^[21]^ with a male-to-female ratio of 4:1, resulting in numbers of 1 in 42 boys and 1 in 189 girls.^[21]^ Despite significant research, the cause and the precise pathophysiology of this glymphatic system dysfunction remain largely unknown.

Currently, the mechanism underlying ASD remains unclear. However, previous studies have demonstrated that dilation of the Virchow-Robin space (VRs) in the cerebral convexities is increased in the ASD group and associated with developmental delay,^[22]^ thus it may have clinical significance.^[23,24]^ Therefore, this study aimed to compare the glymphatic system function in ASD patients to that in healthy controls using the DTI-ALPS method. However, no previous study has investigated the relationship between VRs and ASD in children. Moreover, it is uncertain whether VRs alterations show a correlation with age. To validate changes in glymphatic system function, the correlations of bilateral ALPS index of children with ASD were studied. We hypothesized that ASD has severe glymphatic system malfunction and that the ALPS index is positively related to age. The findings from this study will change the understanding of the mechanism of the disease.

## 2. Materials and Methods

The local institutional review board, the Clinical Research Ethics Committee of Yan’an University, Affiliated Hospital (No. YUSMAFEC2017RC-026), examined and approved this retrospective case–control study. The children’s parents were aware of the potential risks of an MRI examination, such as loud noise, as well as the side effects of oral chloral hydrate. The parents had given written informed consent.

### 2.1. Participants

**Figure 1.**
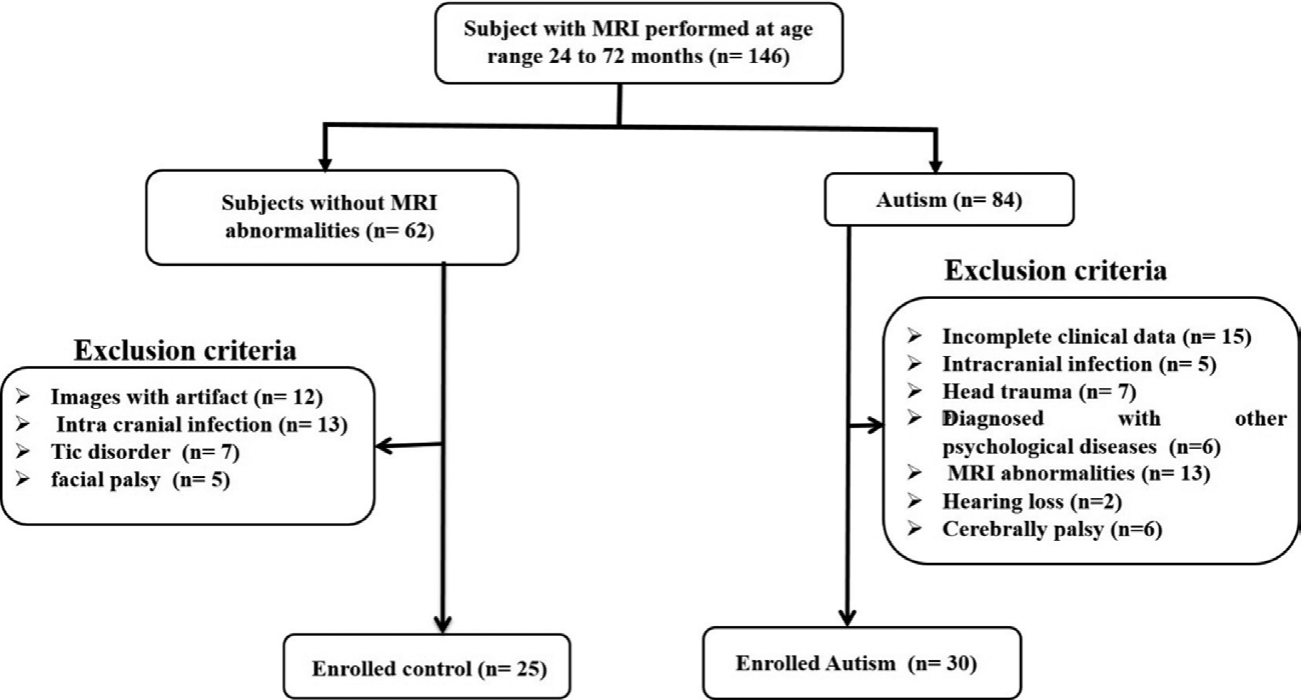
Study flow chart based on inclusion and exclusion criteria.

Patient participants diagnosed with ASD, who completed MRI examinations as part of the screening for brain disorders, were sampled for the present study. Participants were recruited from a consecutive case series of children (aged 24–72 months) assessed at the Neuropsychiatric Child Unit of the Yan’an University, Affiliated Hospital, over the course of 4 years (2017–2021). Inclusion criteria were: clinical diagnosis of ASD, according to Diagnostic and Statistical Manual of Mental Disorders, Fourth Edition, Text Revision (DSM-IV-TR) criteria of the American Psychological Association (APA 2000);^[25]^ gestational age of 37 weeks or more; and no history of brain injury, head trauma, or central nervous system infections. The total number of families invited to participate was 84, and they gave informed consent. The following were the criteria for exclusion: incomplete clinical information; intracranial infection; head trauma; diagnosed with other psychological diseases; MRI abnormalities, such as hyperintensity on T2 fluid-attenuated inversion recovery (FLAIR) (except for the peritrigonal terminal zone of white matter myelination, which appears as a bilaterally symmetrical, slightly increased signal intensity with a hazy border on T2WI);^[26]^ hearing loss; and cerebral palsy. The following criteria were used to enroll children in the control group: children with a gestational age of 37 weeks or more; There is no history of seizures; and there are no MRI abnormalities. Children whose images revealed artifacts were not allowed to participate. Children with neurological problems (such as facial palsy and tic disorders) and intracranial infection were also excluded from the study, as shown in Fig. 1.

### 2.2. MRI data acquisition

At the Yan’an University Affiliated Hospital, all subjects completed MRI examinations using the same 3.0-T scanner (Signa HDxt, GE Healthcare, Milwaukee, WI) with an 8-channel head coil. All children were adjusted while sleeping to eliminate motion artifacts and facilitate the MRI examination. Hearing protection was provided by micro-earplugs, while head immobilization was provided by molded foam. The 3.0-T scanner was used to perform 3-dimensional rapid spoiled gradient-recalled echo T1-weighted imaging (T1WI), fast spin-echo (FSE) T2-weighted imaging (T2WI), T2 FLAIR, and single-shot echo-planar DTI. The parameters of MRI sequences (T1WI, T2WI, and T2-FLAIR) were the same for all participants. T2WI was used to assess VRs, and T1WI and T2-FLAIR were utilized to distinguish VRs from other lesions. The following were the parameters of the MRI sequences: (i) T2WI: TR/TE = 4200/120 ms; slice thickness = 4 mm without gap; field of vision = 240 mm; and matrix size = 320 × 320. (ii) DTI: 30 gradient directions; b values = 0 and 600 s/mm^2^;^[27]^ number of b0 = 8; TR/TE = 11,000/69.5 ms; slice thickness = 2.5 mm without spaces; field of vision = 240 mm; and matrix size = 128.

### 2.3. DTI-ALPS processing and image analysis

The FMRIB software library was used for processing DTI data. First, an eddy current correction was performed for all the DTI of all subjects. Then, the brain regions were extracted using the FMRIB software library Brain Extraction Tool (http://fsl.fmrib.ox.ac.uk/fsl/fslwiki/FDT). Image processing and comparison of ALPS indexes among ASD and control groups were addressed using an optimized protocol as described in previous studies.^[27–29]^

**Figure 2.**
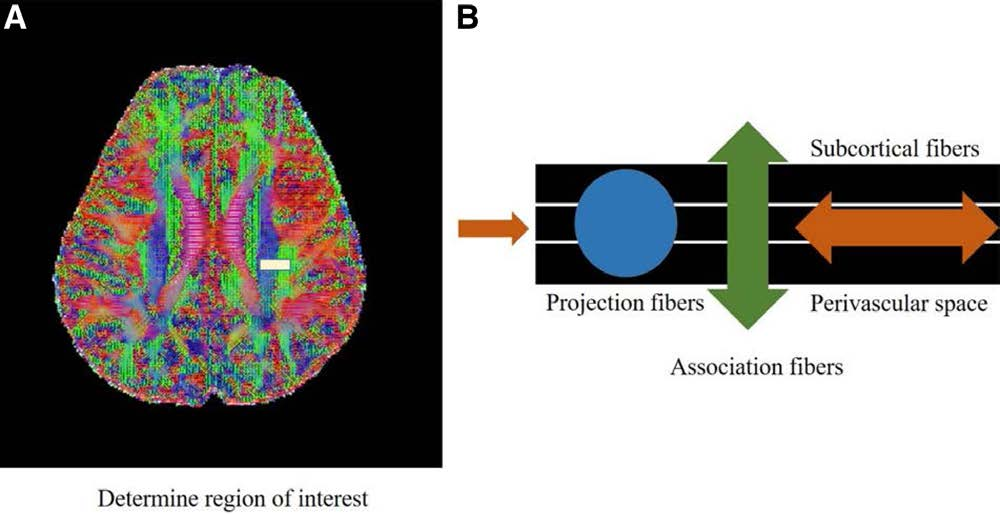
Process illustrating for obtaining diffusion tensor image analysis along the perivascular space (DTI-ALPS) index.

The ALPS index was used to determine the glymphatic system activity. We used the DTI-ALPS processing and measuring method from prior publications.^[11,15,30–33]^13k software were used to create diffusion metric images.^[34]^ The software generates diffusion tensor computational images, which include a color-coded fractional anisotropy map and diffusivity map, as well as the ability to calculate diffusivity in the x-, y-, and z-axes on each image. The slice at the level of the lateral ventricle body was chosen (Fig. 2A). The perivascular space lies perpendicular to the ventricle wall at this level, and so typically runs in the right-left direction (x-axis) on the axial plane. The direction is also perpendicular to the projection fibers’ (mainly in the z-axis) and association fibers’ (primarily in the x-axis) directions (mostly in the y-axis). As a result, the diffusivity along the x-axis at projection/association fiber regions will, at least partially, represent the diffusivity along with the perivascular space. In the left hemisphere, a 5-mm diameter spherical region of interest was placed in the area of the projection fibers, the association fibers, and the subcortical fibers (Fig. 2B). The index value was derived from the ratio of the 2 diffusivity value sets that are perpendicular to the main fibers in the tissue; that is, the ratio of the average values of the x-axis diffusivity in the area of the projection fibers (Dxproj) and the x-axis diffusivity in the area of the association fibers (Dxassoc) to the average value of the y-axis diffusivity in the area of the association fiber. ALPS index = (mean Dxproj, Dxassoc)/ (mean Dxproj, Dxassoc) (Dyproj, Dzassoc).

### 2.4. Statistical analysis

To compare demographic data between ASD and the control group, the Mann–Whitney *U* test was performed. The associations between age and bilateral ALPS-indices were investigated using Pearson correlations. For statistical analysis, IBM’s SPSS software (Version 21.0; Armonk, New York, NY) was utilized. *P < *.017 was determined to be statistically significant using the Bonferroni correction, and multiple comparisons were made across the groups.

## 3. Results

### 3.1. Demographic characteristics

The hospital-based study eventually enrolled 55 patients based on the inclusion and exclusion criteria: 30 in the ASD group and 25 in the control group. ASD and control groups had no significant differences in age, sex, or gestational age (Table 1).

**Table 1:** Demographics for the autism and control groups.

	Autism (n = 30)	Control (n = 25)	*P* value
Age (months)	47.83 ± 15.56	44.80 ± 15.01	.520
GA (wk)	39.67 ± 0.99	39.48 ± 1.16	.596
Gender (male)	24(80.00 %)	18(72.00 %)	.490

GA = gestational age, mean ± standard deviation.

Between patients with ASD and healthy control, there were significant differences in DTI-ALPS index along the z-axis along projection fibers and the y-axis along association fibers (Table 2). The index along the z-axis in the projection fiber and index along the y-axis in the association fibers in patients with ASD were lower than those in the healthy controls on the left (1.02 ± 0.12 vs 1.27 ± 0.25, *P* < .001) index and in the right index (1.03 ± 0.12 vs. 1.32 ± 0.20, *P* < .001). The bilateral index in patients with ASD was significantly lower than in healthy controls.

**Table 2:** Shows bilateral diffusion tensor image analysis along the perivascular space (DTI-ALPS) index among the autism and control groups.

	Autism (n = 30)	Control (n = 25)	*P* value
ALPS—index (left)	1.02 ± 0.12	1.27 ± 0.25	<.001
ALPS—index (right)	1.03 ± 0.12	1.32 ± 0.20	<.001

### 3.2. Correlation analysis between bilateral DTI-ALPS and age

**Figure 3.**
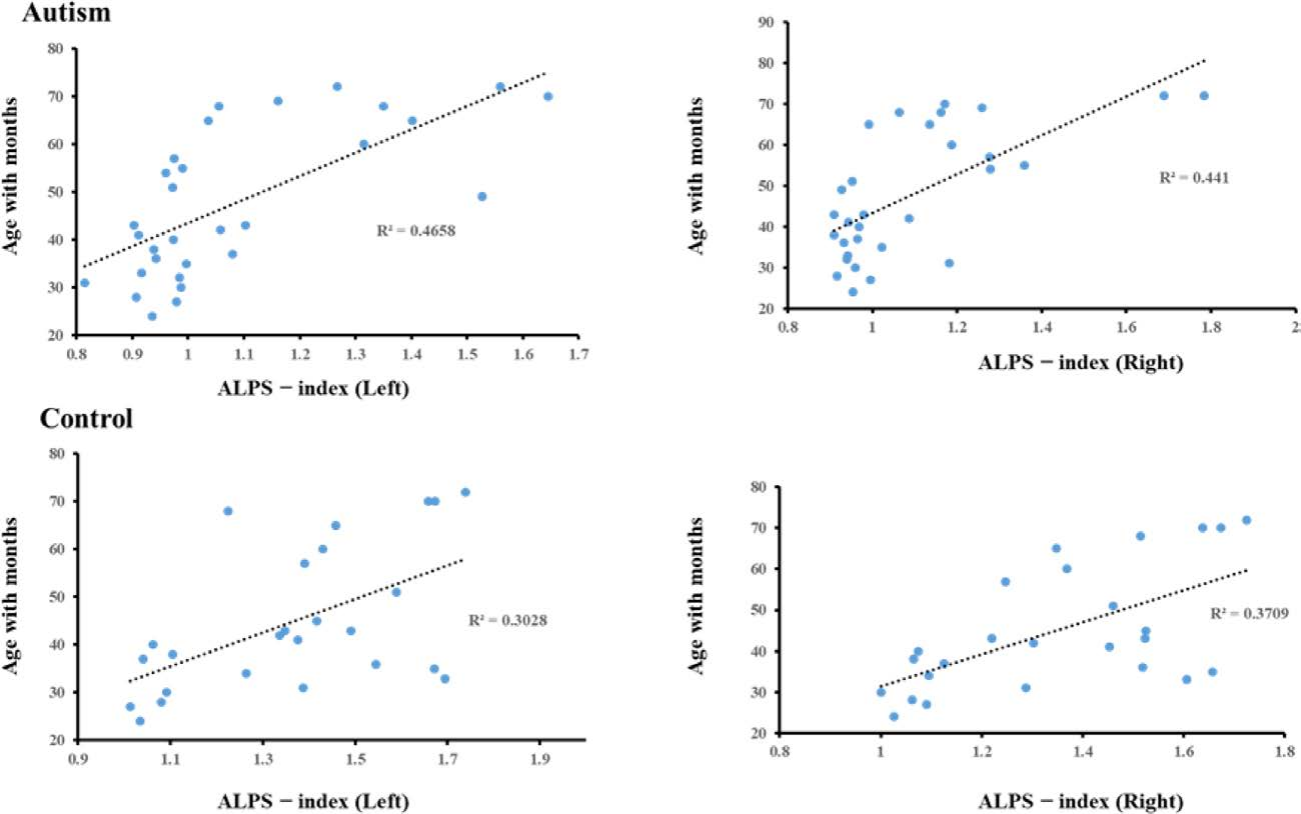
Correlation between bilateral diffusion tensor image analysis along perivascular space index (DTI-ALPS) and age in patients with autism and control groups.

The DTI-ALPS index was significantly positively correlated with age in ASD (left R2 = 0.4658, right R2 = 0.441), and in control (left R2 = 0.3028, and right R2 = 0.3709) as shown in Fig. 3.

## 4. Discussion

In this study, we found that patients with ASD had significantly lower ALPS index than healthy controls, suggesting that patients with ASD have glymphatic system dysfunction. In addition, ALPS index was positively correlated with age in patients with ASD, which demonstrates that the glymphatic system function declines in patients with ASD. Our hypothesis was confirmed by these findings.

Many investigations on neurological illnesses have recently been undertaken as a result of the growing interest in the glymphatic system. Reduced amyloid clearance is linked to the pathogenesis of Alzheimer’s disease, and phosphorylated Tau clearance is linked to chronic traumatic encephalopathy.^[8]^ In the ASD brain, poor interstitial solute clearances and aberrant CSF-ISF communication can be seen.^[6]^ As a result of the breakdown of blood–brain barrier (BBB) integrity caused by an ictal event, serum solute shifts locally in the brain parenchyma, and changes in ISF ionic composition cause water retention.^[35]^ Long-term pathological alterations may alter ISF flow, resulting in a slowed clearance system and hazardous chemical buildup in the brain.^[36]^ Phosphorylated tau is dispersed across cortical neurons, perivascular areas around penetrating pial arteries, and meninges in ASD patients, and extracellular phosphorylated tau is also distributed parallel to venules.^[6]^ The distribution of phosphorylated tau in the brain differs from that of extravasation markers such as immunoglobulin G or albumin. Furthermore, perivascular phosphorylated tau does not extend radially from the vessel but rather follows longitudinal para-vascular tracks, implying that high phosphorylated tau is associated with inefficient waste clearance from the brain.^[36]^

In numerous neurodegenerative illnesses, the DTI-ALPS approach was utilized to assess glymphatic system function. A substantial association between the glymphatic and the VRs volume and counts was discovered in a study of patients with epilepsy and simple febrile seizures.^[27,37]^ In this study comparing the DTI-ALPS index between patients with ASD and control, the ASD group had a lower DTI-ALPS index than the control group. A recent study looked at the DTI-ALPS index in epileptic patients and found that individuals with epilepsy who also had moderate cognitive impairment or dementia had a considerably lower DTI-ALPS index than healthy controls.^[38]^ The DTI-ALPS index was considerably lower in patients with temporal lobe epilepsy than in healthy control.^[16]^ As a result, it’s important to consider the possibility of using a novel biomarker to noninvasively evaluate glymphatic function in patients with ASD.

Using the DTI-ALPS method, previous studies on epilepsy have shown that patients with juvenile myoclonic epilepsy, temporal lobe epilepsy and focal epilepsy have significant glymphatic system dysfunction.^[16,39,40]^ Previous studies have found broad changes in cortical thickness, subcortical volumes, and cerebral white matter in epileptic patients. Furthermore, the changed patterns varied among epileptic syndromes.^[41–43]^ An increased number of activated microglia was found in postmortem brain tissue from epileptic individuals, with enhanced fractions of microglia differing depending on cortical thickness. More research is needed to look into the changes in glymphatic system function in ASD. In this study, the DTI-ALPS index showed a significant positive correlation between age in patients with ASD and healthy controls. Glymphatic system impairment in ASD has been linked to loss of perivascular aquaporin-4 polarization and BBB malfunction.^[6]^ The aggregation of misfolded proteins in numerous neurodegenerative illnesses exacerbates the impaired distribution of growth factors, neuromodulators, carrier proteins, and other solutes due to glymphatic system malfunction. The effect of seizure length on the glymphatic system may be connected to changes in BBB distribution and clearance in the brain parenchyma, as previously indicated.^[36]^ Thus, we predicted that the DTI-ALPS index would differ depending on the duration of ASD, among other clinical parameters; instead, the DTI-ALPS index was significantly inversely correlated with age. However, we find significant differences in the DTI-ALPS index between patients with ASD and healthy controls, suggesting that there are changes in glymphatic system function in ASD. This finding was inconsistent with the finding from a previous study showing glymphatic system dysfunction in patients with focal epilepsy.^[40]^ This difference may come from the fact that we only included patients with ASD in the fast-developing brain age range, while previous studies did not include such an age range. We interpret the positive correlation of DTI-ALPS index and age as potential signs of global DTI parameters associated with poor glymphatic system function in our patients. This is the first study to investigate glymphatic system function in children with ASD. However, this work has some limitations, although we were able to demonstrate glymphatic system failure in ASD. For instance, the sample size was tiny, and the study was not multicentric. In the scan protocol, we used a relatively low b value (600 s/mm^2^), which may have difficulties to differentiate between DTI-ALPS and CSF. Also, we manually drew the ROIs and measured diffusivity along the axis at each of the 3 fibers, which could have resulted in differences across measurements. However, initially, we used a different hue of DTI to identify each fiber. Furthermore, we collected fiber orientation and diffusivity at the voxel level at the region of interest, and then chose one voxel for each fiber with maximum orientation on the same x-axis, potentially improving the accuracy of fiber detection.

## 5. Conclusions

We was able to demonstrate glymphatic system dysfunction in patients with ASD. Furthermore, glymphatic system malfunction is intimately correlated to age. Thus, our findings help to understand how DTI-ALPS can be used as a potential biomarker in ASD to increase the probability of diffusion alterations, emphasizing the necessity of their therapy. Our findings suggest that the DTI-ALPS approach may help assess the function of the glymphatic system in ASD.

## Acknowledgments

We appreciate the contribution of our patients and their families for participating in this study.

## Author contributions

**Conceptualization:** Xin Li.

**Methodology and writing and original draft:** Cailian Ruan.

**Editing:** Abdoulaye Issotina Zibrila.

**Review:** Mazen Musa.

**Software:** Yifan Wu.

**Validation:** Zhang Zhengxiang.

**Funding acquisition:** Heng Liu.

**Supervision and final validation:** Mustafa Salimeen.
